# Genome Sequence of *Coxiella burnetii* Strain AuQ01 (Arandale) from an Australian Patient with Acute Q Fever

**DOI:** 10.1128/genomeA.00964-14

**Published:** 2014-10-02

**Authors:** Mathias C. Walter, Gemma A. Vincent, John Stenos, Stephen Graves, Dimitrios Frangoulidis

**Affiliations:** a Institute of Bioinformatics and Systems Biology, Helmholtz Zentrum, Munich, German Research Center for Environmental Health (GmbH), Neuherberg, Germany; b Department of Genome-Oriented Bioinformatics, Center of Life and Food Science, Weihenstephan, Technische Universität München, Freising, Germany; c Australian Rickettsial Reference Laboratory, Barwon Health, Geelong, Victoria, Australia; d Department of Microbiology, Pathology North-Hunter, NSW Health Pathology, John Hunter Hospital, HRMC, Australia; e Bundeswehr Institute of Microbiology, Munich, Germany

## Abstract

*Coxiella burnetii* strain AuQ01 was isolated from the serum of an Australian acute Q fever patient and represents the first whole genome from this historical Q fever country. This new genome shows distinct differences from existing genomic data and will enhance the understanding of this query pathogen.

## GENOME ANNOUNCEMENT

*Coxiella burnetii*, a Gram-negative coccobacillus belonging to the class *Gammaproteobacteria*, is the causative agent of the zoonosis Q fever. It has a worldwide distribution but the disease is rarely reported, a situation that probably results from the diagnostic challenges presented by *C. burnetii* and lack of requirement for notification of cases in many countries (1). Despite this, Q fever persists with a large reservoir among multiple species, regularly leading to smaller outbreaks.

Since the first publication of the complete genome sequence of the historical Nine Mile strain (2, 3) in 2003, more isolates have been sequenced and published (4–6). Therefore, efforts to enlarge genomic knowledge are important to achieve more information about this pathogen. Although Australia was also the first country in which the Q fever agent was discovered (7), no genome sequence has been available from this continent until now.

Here, we present a strain, initially named Arandale, which was isolated in a cell culture in 2005 from the buffy coat (EDTA blood sample) of a 56-year-old male patient with acute Q fever who lived in Deepwater, New South Wales, Australia (8). Genomic DNA was extracted using MagNA Pure compact system (Roche Diagnostics, Mannheim, Germany). A sequencing library was prepared, with an adapted NEBNext protocol for small amounts of input DNA, and was sequenced by GATC (Constance, Germany) on an Illumina HiSeq 2500 instrument with 2 × 100 paired-end reads.

Sequence reads were assembled using SPAdes (9). The majority of the gaps in the resulting assembly correspond to the positions of insertion sequence (IS) elements, mostly of type IS*1111*. IMAGE (10) was used to extend the contigs at the IS ends and Mauve (11) to order them using *C. burnetii* CbuK_Q154 as the reference genome (NCBI reference sequence no. NC_011528 and NC_011526). Some curation and scaffolding at known conserved IS sites (IS*30* and IS*As1*) and the duplicated region in the plasmid was done manually. Polishing was done using mapping with Mira (12) followed by variation analysis. Gene calling and functional annotation was performed using the PEDANT system (13).

The *C. burnetii* AuQ01 genome assembly consists of 66 chromosomal contigs of about 2,073,000 bp and a QpRS plasmid of 39,269 bp. The numbers of nearly full-length IS elements were determined to be 55 of type IS*1111*, 5 of type IS*30*, and 3 of type IS*As1*.

A whole-genome comparison with all five complete reference genomes revealed strain Q154 as the most similar strain. *In silico* typing of strain AuQ01 showed the same *adaA* deletion variant 1 (14), but multispacer sequence typing (MST) and multiple-locus variable number of tandem repeat (VNTR) analysis (MLVA) generated profiles different from those for Q154 (15).

This short comparison demonstrates that *C. burnetii* has a distinct and different evolution on the Australian continent. Furthermore, this new whole-genome sequence enhances the existing genomic data for *C. burnetii* and is therefore beneficial for genomic studies and analyses in the future.

### Nucleotide sequence accession number.

The whole-genome shotgun project of *C. burnetii* AuQ01 has been deposited at DDBJ/EMBL/GenBank under the accession number JPVV00000000.
